# The Reliability of Human Evaluation of Large Language Models in Health Care Settings: Scoping Review

**DOI:** 10.2196/98184

**Published:** 2026-08-19

**Authors:** Euijun Yang, Siyeon Ko, Hyekyung Woo

**Affiliations:** 1 Department of Health Administration College of Nursing & Health Kongju National University Gongju, Chungcheongnam-do Republic of Korea; 2 Institute of Health and Environment Kongju National University Gongju, Chungcheongnam-do Republic of Korea

**Keywords:** large language models, artificial intelligence, human evaluation, evaluation framework, reliability, health care, scoping review

## Abstract

**Background:**

Integration of large language models (LLMs) into health care has accelerated rapidly, yet reliability concerns pose potential risks to patient safety. Although human evaluation has been widely used as an important approach for assessing LLM reliability, a systematic understanding of how such evaluations have been operationalized across studies remains limited.

**Objective:**

This study aimed to characterize the current landscape of human evaluation frameworks for LLM reliability in health care and to identify similarities and differences between the clinical and public health domains.

**Methods:**

In line with the PRISMA-ScR (Preferred Reporting Items for Systematic Reviews and Meta-Analyses Extension for Scoping Reviews) guidelines, PubMed, Web of Science, the Cochrane Library, CINAHL, and Google Scholar were searched for studies published from January 2016 to July 2025. Eligible studies were English-language original research conducted in health care settings that assessed the reliability of LLM-generated responses through human evaluation. Key exclusion criteria were studies without human evaluation and studies focused primarily on LLM model selection, performance optimization, or technical development. Extracted data were analyzed across 3 dimensions: what was evaluated, who evaluated, and how evaluation was conducted. Reported methodological limitations were also categorized and compared between the clinical and public health domains.

**Results:**

Of the 4347 records identified, 71 studies were included in the final analysis (clinical, n=26; public health, n=45). Six reliability indicators were used: accuracy, relevance, completeness, clarity, safety, and consistency. The clinical domain more frequently assessed guideline concordance, internal consistency, and structural coherence, whereas the public health domain more frequently assessed understandability, harm potential, and repeat response consistency. Single-specialty clinicians were the most common evaluators in both domains, although mixed evaluator panels were observed only in the public health domain. Evaluator panels generally consisted of 5 or fewer members. Five-point Likert scales and researcher-defined rubrics were commonly used evaluation approaches in both domains. Key methodological limitations included evaluator subjectivity, nonstandardized indicators, and limited evaluation scope and settings.

**Conclusions:**

To our knowledge, this is the first review to systematically examine how human evaluations of LLM reliability have been conducted across health care. The focus of reliability evaluation differed across domains, with clinical evaluations giving relatively greater attention to clinical validity and logical rigor, whereas public health evaluations gave relatively greater attention to understandability, practical use, and safe use. These differences suggest that the reliability of health care LLMs is difficult to evaluate adequately using a single universal standard. In addition, the methodological limitations identified in this review indicate that current human evaluation approaches are insufficiently standardized. Therefore, future evaluations of health care LLM reliability need to be guided by standardized evaluation frameworks that reflect domain-specific contexts and encompass indicator definitions, judgment criteria, evaluator guidance, and evaluation procedures.

## Introduction

### Rationale

Large language models (LLMs) are AI technologies that generate human-like natural language responses by learning vast amounts of text data, and their applications have been rapidly expanding across various fields [1]. In the health care domain, the potential use of LLMs is under active consideration, including for clinical decision-making support and provision of health information [2,3]. LLMs potentially enhance the accessibility and use of health information not only for health care professionals but also for patients and the general public [4]. However, concerns regarding the reliability of LLMs have also increased alongside these potential applications. Recent studies have reported that LLMs may generate recommendations inconsistent with clinical guidelines and provide inconsistent information due to hallucinations and variability across responses [5-8]. In addition, the possibility that LLMs may provide inappropriate or unsafe medical advice has been empirically demonstrated [9], suggesting that these reliability concerns may extend beyond information quality and contribute to inappropriate health behaviors, distorted clinical judgment, and increased patient safety risks [7,10,11]. Accordingly, systematic evaluation frameworks are increasingly needed to assess the reliability of LLM-generated responses in health care [12].

In health care settings, LLM applications span both the public health and clinical domains, which serve distinct purposes and populations [13,14]. In the public health domain, LLMs provide health information, support patient education, and facilitate health counseling for patients and the general public, primarily supporting disease prevention and health promotion. In the clinical domain, LLMs support physician decision-making in terms of diagnosis, treatment, and examination, and assist clinical research and clinical documentation in contexts directly related to patient care. The two domains differ in terms of service objectives, risk levels, scope of accountability, and regulatory environments [15], suggesting that the reliability evaluation criteria should also be contextually differentiated. However, prior studies have tended to evaluate LLM reliability without sufficiently considering the domain-specific characteristics, thereby limiting the interpretability and applicability of the findings [16,17].

The reliability of LLM-generated responses is primarily verified via both benchmark and human evaluation. Although the former enables objective comparisons based on the correspondence with predefined “correct” answers [18], it does not fully capture the contextual appropriateness and practical use required in real-world health care [19,20]. Consequently, human evaluation, in which human evaluators directly assess the trustworthiness of LLM-generated responses based on one or more evaluation indicators, has been increasingly recognized as a core method for evaluation of LLM reliability in the health care field [21,22]. Although prior reviews have examined LLM evaluation in health care, they have primarily focused on application areas and performance outcomes [15,22]. However, studies that have systematically examined how human evaluation was conducted in practice, including the indicators used, evaluators involved, and evaluation procedures, remain limited.

Therefore, this review aimed to conduct a scoping review of studies in which humans evaluated the reliability of LLM-generated responses in health care. Specifically, we systematically analyzed the evaluation indicators, evaluator characteristics, and evaluation approaches and examined differences between the clinical and public health domains. In addition, we synthesized the methodological limitations reported in the included studies and identified key considerations for the future development of reliability evaluation frameworks. These findings are expected to provide foundational evidence to support the standardization of LLM reliability evaluation and improve comparability across studies in health care contexts.

### Objectives

This review posed the following research questions:

1. What evaluation indicators, evaluator characteristics, and evaluation approaches have been used in human evaluation-based LLM reliability evaluations in the health care domain?
2. How do these evaluation frameworks differ between the clinical and public health domains?
3. What limitations are commonly observed in terms of human evaluation-based LLM reliability evaluations?

## Methods

### Protocol and Registration

This review is a scoping review of studies that used human evaluators to evaluate the reliability of LLM-generated responses in the health care domain. The review adhered to the PRISMA-ScR (Preferred Reporting Items for Systematic Reviews and Meta-Analyses Extension for Scoping Reviews) guidelines to enhance transparency and reproducibility in reporting [23]. Search reporting was additionally informed by the PRISMA-S (Preferred Reporting Items for Systematic Reviews and Meta-Analyses Extension for Searching) recommendations to strengthen the transparency and reproducibility of the search process [24]. The PRISMA-ScR reporting checklist is shown in Multimedia Appendix 1. A formal review protocol was not publicly registered.

### Information Sources

A systematic literature search was conducted separately in PubMed, Web of Science, the Cochrane Library, and CINAHL. Google Scholar was used as a supplementary source to screen the first 300 relevance-ranked results for arXiv preprints, consistent with previous recommendations [25]. The publication period was restricted from January 1, 2016, to July 31, 2025. The final search was conducted on June 29, 2026, and no subsequent search update was performed. To further improve search comprehensiveness, backward and forward citation searching was conducted for the included studies.

### Search

The search strategy was developed with reference to the key search terms and search methods used in previous studies on LLMs, generative AI, and health care [15,26,27]. Search terms were organized based on the Population, Concept, and Context framework recommended for scoping reviews [28]. As no specific participant population was required, the Concept was defined as human evaluation of LLM reliability and the context as health care settings. The search terms were grouped into 4 core concepts: LLMs, evaluation, reliability, and health care. Controlled vocabulary and free-text terms were used as appropriate for each source and combined using Boolean operators. Only a publication date restriction was applied during the searches, with no additional restrictions based on study design or publication type. The search strategy was iteratively reviewed and refined through discussions among the research team. The full search strategies for each database are provided in Multimedia Appendix 2.

### Eligibility Criteria

Eligibility criteria were defined according to the review objective (Textbox 1). Original studies published in English that assessed, through human evaluation, the reliability of responses generated by LLMs or LLM-based chatbots in health care were included, provided that the full text was accessible. Studies that conducted both human and automated evaluation were included when human evaluation results were reported separately, but the analysis in this review was limited to human evaluation data.

Studies that did not conduct human evaluation of LLM-generated responses, studies primarily focused on LLM model selection or performance optimization, and studies focused on the development of LLMs or related technologies themselves were excluded.

**Eligibility criteria.**

Inclusion criteria

- Studies conducted in the health care domain, encompassing both clinical and public health contexts
- Original studies that assessed the reliability of large language model (LLM)–generated responses via human evaluation, including studies using both human and automated evaluation if human evaluation results were reported separately
- Studies published in English with accessible full texts

Exclusion criteria

- Studies that were not original research (eg, reviews, editorials, commentaries, perspectives, or opinion papers)
- Studies that did not involve human evaluation of LLM-generated responses (eg, benchmark-only studies or studies using automated performance evaluation alone)
- Studies primarily focused on selecting or optimizing LLMs for specific tasks
- Studies primarily focused on the technical development of LLMs or related technologies without evaluating the reliability of LLM-generated responses in health care
- Studies published in languages other than English or for which the full text was unavailable

### Selection of Sources of Evidence

Two researchers (EY and SK) independently conducted the study selection process using Rayyan (Rayyan Systems), including title and abstract screening and full-text review, after duplicate removal [29]. After initial screening based on titles and abstracts, full texts were reviewed for eligibility. Any discrepancies were resolved through discussion and consensus between the 2 reviewers.

### Data Charting Process

Data from the final included studies were systematically extracted and recorded using a Microsoft Excel spreadsheet. One researcher (EY) initially extracted the data, and another researcher (SK) subsequently reviewed the extracted data for accuracy and consistency. The data charting form was developed by the research team based on the review objective and was iteratively reviewed and refined during the review process. When the classification of the domain, application area, or other data items was unclear, the 2 researchers (EY and SK) classified the items by considering the study objectives, the context of LLM use, and other relevant information reported in the full text. Any discrepancies were resolved through discussion and consensus.

### Data Items

The extracted data included title, study design, publication year, first author, country of the first author, LLM model, LLM task, domain, application area, evaluation indicators, evaluator characteristics, evaluation approaches, and reported limitations. The complete dataset is presented in Multimedia Appendix 3. To further analyze human evaluations of LLM reliability, an analytical framework centered on the core components of evaluation was established. The framework comprised 3 analytical dimensions: what was evaluated (evaluation indicators), who conducted the evaluation (evaluator characteristics), and how the evaluation was performed (evaluation approaches). The operational definitions and key examples for each dimension are presented in Table 1.

**Table 1:** Core dimensions and operational definitions used to analyze human evaluation of large language model reliability in health care settings.

Category	Definition	Key examples
Evaluation indicator	Specific dimensions or attributes of LLM^a^-generated responses used to assess reliability	Accuracy, clarity
Evaluator characteristics	Characteristics of the human evaluators involved in assessing reliability, including their expertise and professional roles	Clinicians, nurses, researchers
Evaluation approach	Methods or tools used to measure evaluation indicators	Likert scales, researcher-defined rubrics

^a^LLM: large language model.

### Critical Appraisal of Individual Sources of Evidence

Quality of the identified studies was not assessed as this is not a requirement of scoping reviews.

### Synthesis of Results

Descriptive analyses involving the calculation of frequencies and percentages were performed using Microsoft Excel. Evaluation indicators were integrated based on conceptual similarities in their names and definitions to derive core indicators and subcriteria. The distributions of evaluation indicators, evaluator characteristics, and evaluation approaches were compared between the clinical and public health domains. Methodological limitations were also categorized and compared across the 2 domains.

## Results

### Selection of Sources of Evidence

**Figure 1:**
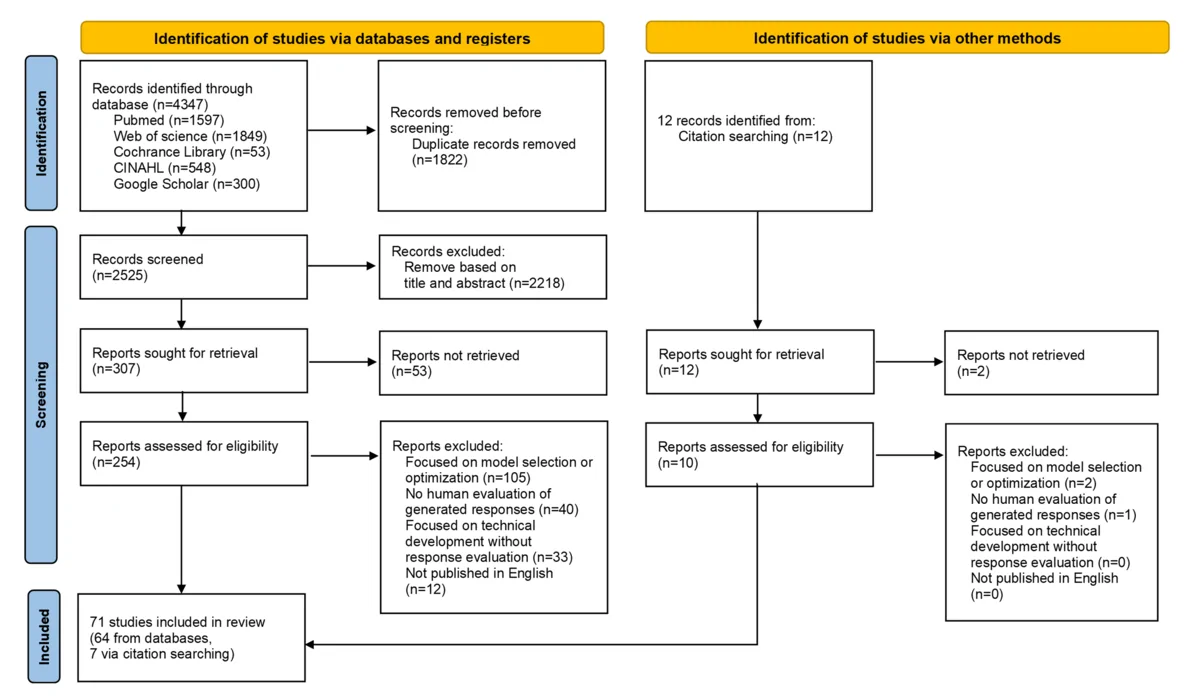
PRISMA-ScR (Preferred Reporting Items for Systematic Reviews and Meta-Analyses Extension for Scoping Reviews) flow diagram illustrating the identification, screening, eligibility assessment, and inclusion of studies through database and citation searching.

A total of 4347 records were identified across the 5 databases. After removing 1822 duplicate records, 2525 underwent title and abstract screening, of which 2218 were excluded. The remaining 307 reports were sought for retrieval, and 53 were not retrieved. A total of 254 reports were assessed for eligibility via full-text review. A total of 190 reports were excluded because they focused on model selection or optimization (n=105), did not include human evaluation of generated responses (n=40), focused on technical development (n=33), or were not published in English (n=12). In addition, 12 records were identified through citation searching; 2 reports were not retrieved, and 3 of the 10 reports assessed for eligibility were excluded. Ultimately, 71 studies [30-100] were included, comprising 64 studies [31-38,41-43,46-59,61-67,69-100] identified through database searching and 7 studies [30,39,40,44,45,60,68] through citation searching. The literature selection process is presented in Figure 1 in accordance with the PRISMA-ScR guidelines.

### Characteristics of Sources of Evidence

**Figure 2:**
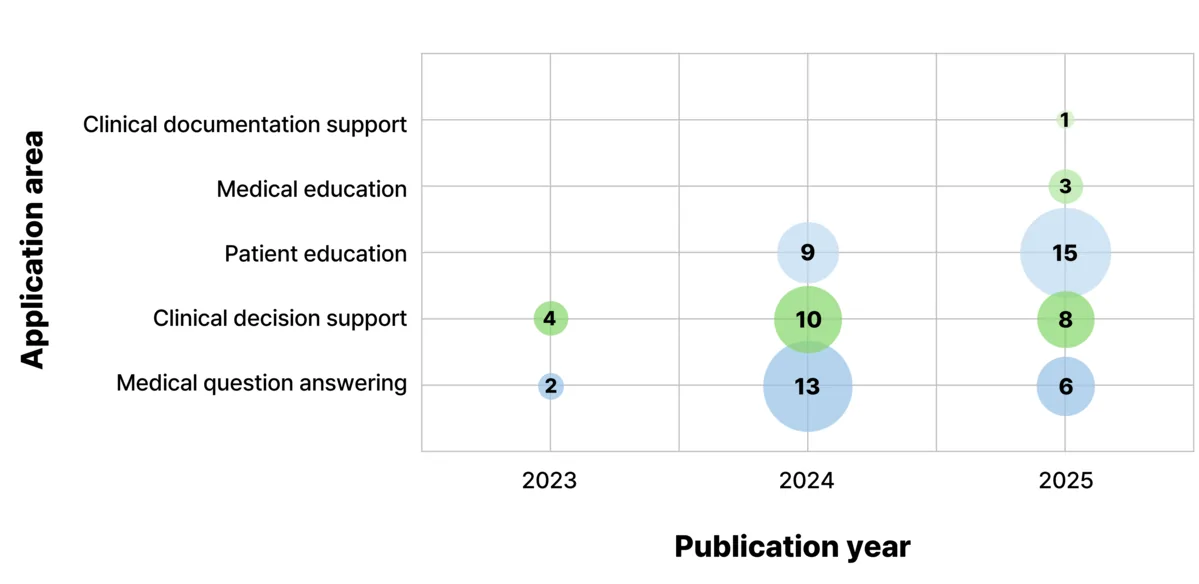
Distribution of the included studies by large language model application area and publication year. Blue circles represent studies in the clinical domain, and green circles represent studies in the public health domain. The number within each circle and the circle size indicate the number of studies.

Of the finally included studies, 26 studies (36.6%) [30-45,75-80,84,86,96,97] were classified under the clinical domain and 45 studies (63.4%) [46-74,81-83,85,87-95,98-100] under the public health domain. In terms of publication year, 6 studies (8.5%) [35-37,41,62,72] were published in 2023, 32 studies (45.1%) [30,32,33,38-40,46,48,51,52,58,60,61,65-70, 74,75,79,82,84,86, 87,90-94,99] in 2024, and 33 studies (46.5%) [31,34,42-45,47,49,50,53-57,59,63,64,71,73,76-78, 80,81,83,85,88,89,95-98,100] through July 2025, indicating a continued increase in health care LLM reliability evaluation research, with 2025 representing a partial year. Although the search covered literature published from 2016 onward, no eligible studies were identified prior to 2023. The LLM application areas were classified into 5 categories based on the literature: medical question answering, clinical decision support, patient education, medical education, and clinical documentation support [101]. Figure 2 presents the distribution of included studies by publication year and LLM application area, illustrating both the increase in study volume and the diversification of application areas over time. In 2023, studies were limited to medical question answering and clinical decision support. In 2024, patient education emerged as an additional application area, while medical question answering and clinical decision support continued to account for a substantial proportion of studies. In 2025, studies covered all 5 application areas, with medical education and clinical documentation support newly identified. Regarding study design, comparative studies accounted for the majority of studies (38/71, 53.5%) [31,36,39-44,47-50,52-55,57,59-61,64,68,76,77,79,81,82,84-91,95,96,99], followed by observational studies (23/71, 32.4%) [33-35,38,46,51,58,62,63,65-67,69-74,78,92-94,100] and diagnostic accuracy studies (10/71, 14.1%) [30,32,37,45,56,75,80,83,97,98]. Studies were conducted across North America, Europe, Asia, and Oceania. The United States contributed the largest number of studies (16/71, 22.5%) [30,36,43,44,51-53,63,66,68,75,79,83,84,92,95], followed by Türkiye (12/71, 16.9%) [35,49,57,59,62,71,72,74,91,94,96,98], China (9/71, 12.7%) [31,32,48,50,61,64,69,97,99], and Germany (7/71, 9.9%) [33,34,38,41,60,87,88]. In terms of the LLM models evaluated, OpenAI’s ChatGPT was assessed in nearly all studies (70/71, 98.6%) [30-55,57-100]. Google’s Gemini/Bard/SLE models were evaluated in 25 studies (25/71, 35.2%) [31,40,44,48-50,52,53,55-57,59,60,76,77,81, 82,84,86-89,91,95,96], Anthropic’s Claude in 9 studies (9/71, 12.7%) [31,41,48,50,52,77,81,89,95], Microsoft’s Copilot/Bing models in 9 studies (9/71, 12.7%) [40,49,52,53,55,76,85,86,91], and Meta’s Llama-based models in 2 studies (2/71, 2.8%) [44,89]. Detailed characteristics of the included studies are presented in Multimedia Appendix 3.

### Critical Appraisal Within Sources of Evidence

The risk of bias in studies was not assessed, in line with scoping review methodology.

### Results of Individual Sources of Evidence

The detailed results reported by each included study are presented in Multimedia Appendix 3, which summarizes the key data extracted from the included studies.

### Synthesis of Results

#### Evaluation Indicators

Across the 71 included studies [30-100], various evaluation indicators were used to assess reliability; however, their terminology and definitions varied considerably. In this review, reliability was not treated as a fixed a priori construct but was operationally defined as the set of evaluation attributes used by human evaluators to determine whether LLMs could be trusted and appropriately used in health care contexts. To address this terminological heterogeneity and inductively derive the components of reliability, all evaluation indicators used in the included studies were extracted verbatim and organized according to their original names. The frequency of each indicator was then calculated, and the indicators were reviewed in descending order of reporting frequency. Lower-frequency indicators were integrated into higher-frequency indicators when they were conceptually similar or when their definitions fell within the conceptual scope of the higher-frequency indicators. In contrast, indicators that remained specific to particular studies after examination of their definitions, as well as indicators that were too broad to be classified as a single evaluation concept, were categorized as “Other.” The analysis focused on indicators that could be clearly classified. Through this process, 6 core indicators were identified: accuracy, relevance, completeness, clarity, safety, and consistency. The original indicator names and their classification by core indicator are presented in Multimedia Appendix 3. The definitions and evaluation criteria of the indicators integrated into each core indicator were compared and synthesized to derive common subcriteria and corresponding definitions, as presented in Table 2.

Table 3 presents the frequencies of the 6 core reliability evaluation indicators and their subcriteria across the clinical and public health domains. As a single evaluation indicator may encompass multiple subcriteria, the sum of the percentages within each domain exceeds 100%. For example, if a study defined accuracy as a concept that incorporated both guideline concordance and currency, that study was counted under both subcriteria [75]. In addition, 2 studies [96,97] in the clinical domain and 3 studies [98-100] in the public health domain that did not report definitions of the evaluation indicators were classified separately as “Not Reported.”

Overall, accuracy was the most frequently used core evaluation indicator in both domains, although differences were observed in the relative emphasis placed on specific subcriteria. In the clinical domain, guideline concordance was used as a major evaluation component alongside factual correctness, whereas in the public health domain, evaluation primarily focused on factual correctness. Evidence and source validity were assessed at similar frequencies across the 2 domains, while studies explicitly incorporating currency were limited in both domains.

Relevance was assessed primarily via query-response alignment and actionability in the clinical domain, with contextual appropriateness additionally used in a subset of studies. In the public health domain, actionability was the most frequently used subcriterion, followed by query-response alignment.

In terms of completeness, studies in the clinical domain mainly focused on core element coverage, assessing whether responses addressed the clinically required components. In the public health domain, core element coverage was the most frequently assessed subcriterion, followed by information sufficiency, whereas detail inclusion was infrequently assessed.

Clarity was most frequently assessed through understandability, particularly in the public health domain. In contrast, structural coherence was more frequently assessed in the clinical domain than in the public health domain.

In terms of safety, studies evaluating both harm potential and confusion potential were observed in both domains. Both were reported approximately 3 times as frequently in the public health domain as in the clinical domain (harm potential: clinical, 2/26, 7.69%; public health, 10/45, 22.22%; confusion potential: clinical, 1/26, 3.85%; public health, 5/45, 11.11%). Warning provision was assessed in only 1 clinical study and was not assessed in the public health domain.

Patterns also differed for Consistency. Repeat response consistency was more frequently assessed in the public health domain, whereas internal consistency was assessed more than 3 times as frequently in the clinical domain (4/26, 15.38%) as in the public health domain (2/45, 4.44%).

**Table 2:** Core reliability evaluation indicators and subcriteria used for human evaluation of large language model-generated responses in health care settings, derived from 71 studies^a,b,c^.

Evaluation indicator		Definition	Studies	
Accuracy				[30-85]
	Factual correctness	Factually accurate and free from errors or distortions		
	Guideline concordance	Consistent with established clinical guidelines, the medical literature, or health policies		
	Currency	Reflecting up-to-date medical knowledge, public health information, or policy changes		
	Evidence and source validity	Supported by credible and appropriate evidence or references		
Relevance				[30,33-35,37-40,42,44,45,47-50,53,54, 56-58,60,62,63,67,70,71,74-76,78,81,84-90]
	Query-response alignment	Directly addressing the intent of the query		
	Contextual appropriateness	Appropriately tailored to the given context, including user characteristics or situational factors		
	Actionability	Provision of useful and actionable guidance applicable to real-world decision-making or behavior		
Completeness				[35-37,40,42-44,46-48,52,53,55-61, 64,65,68-72,74-77,81,85-88,91,92]
	Core element coverage	Covering all essential components required to address the query		
	Information sufficiency	Provision of sufficient and nonomissive information		
	Detail inclusion	With detailed and in-depth information beyond superficial descriptions		
Clarity				[35,40,42,44,47,49-53,55,57, 58,60,62,63,71,73-76,82,86-90]
	Understandability	Clear and easily understandable by the intended audience		
	Structural coherence	Logically organized and well-structured		
Safety				[37,41,50-52,54-56,65,70,78,79,81,85,90,92-94]
	Harm potential	With information that may pose risks to health or safety		
	Confusion potential	Absence of misunderstandings, confusion, or unnecessary concern because of ambiguity or inaccuracy		
	Warning provision	Provision of appropriate warnings, cautions, or risk mitigation guidance		
Consistency				[32,42,44,46,64,68,69,72,73,75,86,90,95]
	Repeat response consistency	Remaining stable across repeated outputs for the same query		
	Internal consistency	Logically consistent, without internal contradictions		

^a^Indicators and subcriteria were derived through comparative analysis of evaluation frameworks reported across the included studies.

^b^A single study could use more than one evaluation indicator or subcriterion; therefore, studies may be counted in multiple categories.

^c^Reference numbers correspond to studies that used each indicator.

**Table 3:** Core reliability evaluation indicators and subcriteria used in human evaluations of large language models across the clinical and public health domains^a^.

Evaluation indicator		Clinical (n=26)			Public health (n=45)	
		Studies, n (%)	References	Studies, n (%)		References
Accuracy						
	Factual correctness	16 (61.54)	[30-45]	29 (64.44)		[46-74]
	Guideline concordance	7 (26.92)	[45,75-80]	5 (11.11)		[50,54,81-83]
	Currency	2 (7.69)	[37,75]	2 (4.44)		[50,70]
	Evidence and source validity	4 (15.38)	[35,39,75,84]	6 (13.33)		[50,52,62,74,81,85]
Relevance						
	Query-response alignment	9 (34.62)	[33,34,38,40,44,45,75,76,86]	8 (17.78)		[47-49,53,54,56,67,71]
	Contextual appropriateness	4 (15.38)	[30,39,78,84]	4 (8.89)		[53,60,87,88]
	Actionability	7 (26.92)	[30,34,35,37,42,78,84]	13 (28.89)		[49,50,53,57,58,62,63,70,74,81,85,89,90]
Completeness						
	Core element coverage	7 (26.92)	[36,40,42,43,75-77]	16 (35.56)		[48,52,53,55-58,60,64,68,69,71,87,88,91,92]
	Information sufficiency	3 (11.54)	[35,37,44]	10 (22.22)		[46,47,59,61,65,70,72,74,85,92]
	Detail inclusion	2 (7.69)	[76,86]	4 (8.89)		[48,56,81,92]
Clarity						
	Understandability	7 (26.92)	[35,40,42,44,75,76,86]	20 (44.44)		[47,49-53,55,57,58,60,62,63,71,73,74,82,87-90]
	Structural coherence	4 (15.38)	[42,44,75,86]	1 (2.22)		[50]
Safety						
	Harm potential	2 (7.69)	[41,79]	10 (22.22)		[50-52,54,56,65,85,92-94]
	Confusion potential	1 (3.85)	[37]	5 (11.11)		[55,56,70,81,90]
	Warning provision	1 (3.85)	[78]	0 (0)		—^b^
Consistency						
	Repeat response consistency	2 (7.69)	[32,75]	6 (13.33)		[46,64,68,69,72,90]
	Internal consistency	4 (15.38)	[32,42,44,86]	2 (4.44)		[73,95]
Not reported^c^		2 (7.69)	[96,97]	3 (6.67)		[98-100]

^a^Percentages within each domain exceed 100% because a single study could be classified under multiple subcriteria within the same evaluation indicator.

^b^Not available.

^c^Not reported refers to studies that did not provide definitions for the evaluation indicators used.

#### Evaluator Characteristics

Table 4 presents the evaluator compositions and panel sizes used in reliability evaluations across the clinical and public health domains.

In the clinical domain, evaluations involving only single-specialty clinicians accounted for the majority of studies. Panel sizes ranged from 1 to 6 or more evaluators, with panels of 1-2 and 3-5 evaluators being equally the most common. Evaluations involving multispecialty clinicians were identified in only a few studies, and evaluations involving only nonclinicians were limited to 2 studies. Evaluator composition was not clearly reported in 2 studies [42,84].

In the public health domain, evaluator composition was more diverse. Although evaluations involving only single-specialty clinicians remained the most common, mixed evaluator panels comprising both clinicians and nonclinicians were identified only in the public health domain and across multiple studies. The specific compositions varied across studies and included combinations of clinicians, nurses, midwives, postgraduate students, patients, and laypersons [47,66,90]. In addition, an evaluator panel that did not include any clinicians was identified in 1 study.

**Table 4:** Evaluator characteristics in human evaluations of large language models across the clinical and public health domains.

Evaluator characteristics		Clinical (n=26)		Public health (n=45)	
		Studies, n (%)	References	Studies, n (%)	References
Clinicians only (single-specialty)					
	1-2	7 (26.92)	[31,35,40,43,44,76,79]	13 (28.89)	[46,49,50,56,59,61,62,67,69,72,83,91,98]
	3-5	7 (26.92)	[30,33,37,38,45,80,97]	10 (22.22)	[51,52,55,60,63,65,81,85,89,92]
	≥ 6	5 (19.23)	[32,39,75,77,86]	9 (20)	[54,57,71,82,87,88,93,94,99]
Clinicians only (multispecialties)					
	1-2	0 (0)	—^a^	0 (0)	—
	3-5	1 (3.85)	[41]	2 (4.44)	[68,70]
	≥ 6	2 (7.69)	[34,36]	0 (0)	—
Mixed evaluators^b^					
	1-2	0 (0)	—	1 (2.22)	[95]
	3-5	0 (0)	—	3 (6.67)	[53,64,74]
	≥6	0 (0)	—	5 (11.11)	[47,66,73,90,100]
Others^c^					
	1-2	1 (3.85)	[78]	0 (0)	—
	3-5	1 (3.85)	[96]	1 (2.22)	[48]
	≥6	0 (0)	—	0 (0)	—
Not specified^d^		2 (7.69)	[42,84]	1 (2.22)	[58]

^a^Not available.

^b^Mixed evaluators refer to panels comprising both clinicians and nonclinicians within a single study such as patients, caregivers, researchers, or students.

^c^Others refer to panels comprising only nonclinicians such as anatomists or researchers.

^d^Not specified refers to studies in which evaluators were identified as clinicians but their clinical specialties were not reported, or in which the evaluator composition was not reported.

#### Evaluation Approach

Table 5 presents the evaluation approaches used in reliability evaluations across the clinical and public health domains. Because a single study could use multiple evaluation approaches, the sum of percentages within each domain exceeds 100%. For example, if a study used both a 3-point and a 6-point Likert scale, it was counted in both Likert scale categories [100].

In the clinical domain, Likert scale-based evaluation was the most frequently used approach, and the 5-point scale was the most common. Other scale formats included 3-point, 4-point, and 6-point scales. Researcher-defined rubrics were also identified in several studies, whereas validated instruments and checklists were infrequently used. Combined approaches were also identified in a subset of studies.

In the public health domain, Likert scale-based evaluations and researcher-defined rubrics were both commonly used, with the 5-point scale being the most frequently used Likert scale format. In addition to 3-point and 6-point scales, 10-point and 12-point scales were used, indicating greater variation in scale formats than in the clinical domain. Validated instruments and checklists were infrequently used. Combined approaches were more frequently reported than in the clinical domain. Examples included researcher-defined rubrics combined with 5-point Likert scales, 5-point Likert scales combined with the Global Quality Scale (GQS), and researcher-defined rubrics combined with checklists [49,50,65,87].

**Table 5:** Evaluation approaches used in human evaluations of large language models across the clinical and public health domains^a^.

Evaluation approach		Clinical (n=26)			Public health (n=45)	
		Studies, n (%)	References	Studies, n (%)		References
Likert scale						
	3-point	3 (11.54)	[36,39,84]	2 (4.44)		[73,100]
	4-point	1 (3.85)	[77]	0 (0)		—^b^
	5-point	11 (42.31)	[30,33,34,37-39,42,45,75,80,84]	13 (28.89)		[47,48,54-57,67,70,71,74,90,93,99]
	6-point	2 (7.69)	[36,39]	4 (8.89)		[73,90,98,100]
	10-point	0 (0)	—	1 (2.22)		[94]
	12-point	0 (0)	—	1 (2.22)		[98]
Researcher-defined rubric^c^		8 (30.77)	[31,32,35,40,43,76,78,86]	15 (33.33)		[46,52,53,59,61-63,66,68,69,72,82,83,85,95]
Validated instruments^d^		1 (3.85)	[96]	1 (2.22)		[89]
Combined approaches^e^		3 (11.54)	[41,44,97]	11 (24.44)		[49-51,58,60,64,65,87,88,91,92]
Checklists		1 (3.85)	[79]	1 (2.22)		[81]

^a^Percentages within each domain exceed 100% because a single study could be classified under multiple categories when multiple evaluation approaches were used.

^b^Not available.

^c^Researcher-defined rubrics refer to evaluation criteria or scoring frameworks constructed by the researchers for the specific purposes of an individual study.

^d^Validated instruments refer to standardized assessment tools such as DISCERN and the Patient Education Materials Assessment Tool (PEMAT).

^e^Combined approaches refer to the use of 2 or more evaluation methods within a single study, including Likert scales, researcher-defined rubrics, validated instruments, and checklists.

### Methodological Challenges in Reliability Evaluation Across Domains

**Figure 3:**
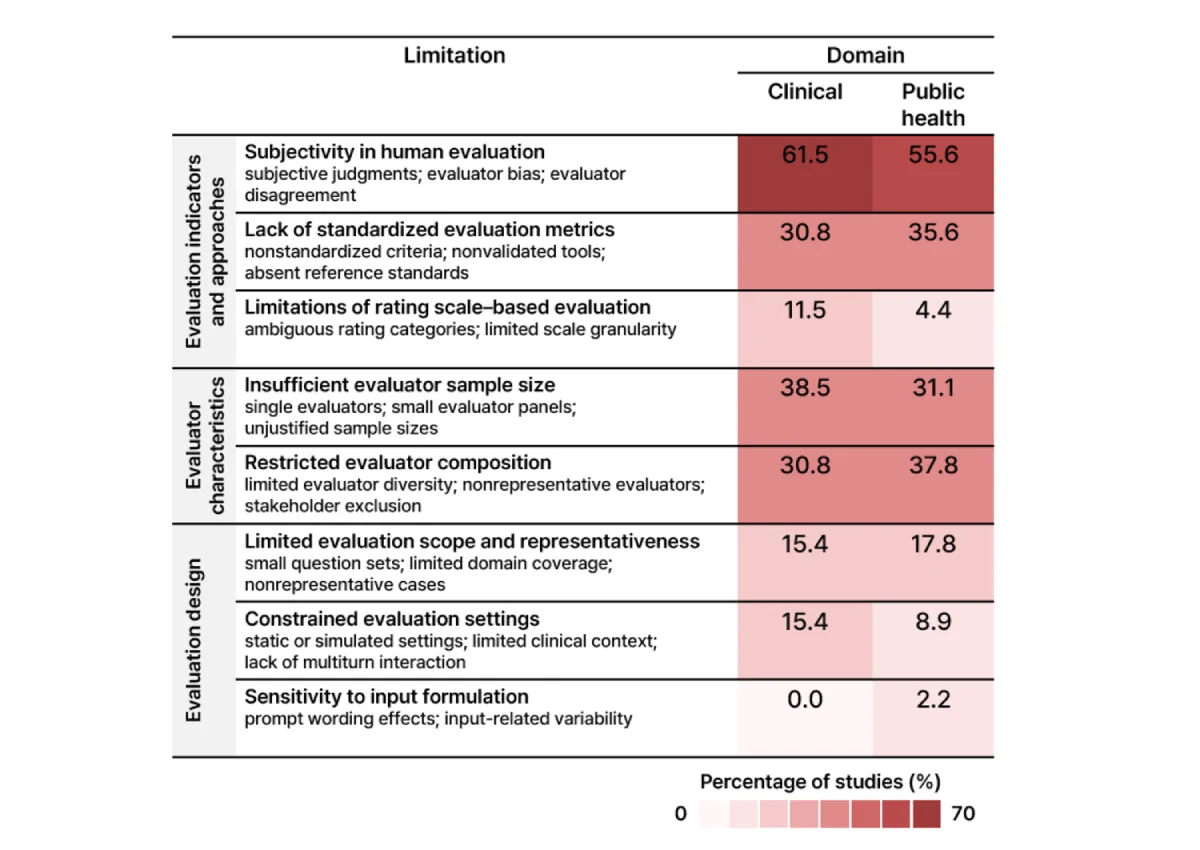
Frequencies of reported methodological limitations in human evaluations of large language model reliability across the clinical and public health domains. Percentages represent the proportion of studies within each domain, and darker colors indicate higher reporting frequencies.

Figure 3 presents the distribution of methodological limitations reported in the clinical (n=26) and public health (n=45) domains, enabling visual comparison of their relative frequencies. Overall, subjectivity in human evaluation was the most frequently reported limitation in both domains. In the clinical domain, insufficient evaluator sample size was the next most frequently reported limitation, whereas restricted evaluator composition and lack of standardized evaluation metrics were relatively common in the public health domain. Insufficient evaluator sample size and limitations of rating scale–based evaluation were reported more frequently in the clinical domain, while restricted evaluator composition and lack of standardized evaluation metrics were more frequently reported in the public health domain. Limited evaluation scope and representativeness were reported at similar frequencies across the 2 domains, while constrained evaluation settings were more frequently reported in the clinical domain. Sensitivity to input formulation was reported in only 1 study in the public health domain. Specific examples of each limitation and a list of relevant studies are provided in Multimedia Appendix 4.

## Discussion

### Principal Findings

We systematically examined the evaluation indicators, evaluator characteristics, and evaluation approaches used in human evaluations of LLM reliability in health care and compared the clinical and public health domains. Reliability evaluation was centered on 6 core evaluation indicators: accuracy, relevance, completeness, clarity, safety, and consistency. Among the subcriteria, guideline concordance, internal consistency, and structural coherence were more frequently assessed in the clinical domain, whereas understandability, harm potential, and repeat response consistency were more frequently assessed in the public health domain. Clinicians constituted the primary evaluator group in both domains, and evaluator panels generally consisted of 5 or fewer members. Likert scales and researcher-defined rubrics were the most commonly used evaluation approaches. These findings provide practical guidance for identifying the key elements that should be considered when evaluating LLM reliability across health care contexts and for improving the methodological quality of human evaluation.

### Domain-Specific Differences in Evaluation Indicators and Conceptualization of Reliability

A key finding of this review was that evaluation indicators for LLM reliability differed between the clinical and public health domains, reflecting differences in how reliability is conceptualized across health care contexts.

In the clinical domain, the validity and contextual appropriateness of LLM responses for real-world clinical judgment were treated as core components of reliability. Because clinical decision-making is influenced by a patient’s symptoms, medical history, comorbidities, and treatment setting, the appropriateness of the same medical information may differ across specific clinical situations [102,103]. Accordingly, reliability in the clinical domain can be understood as extending beyond factual accuracy to include whether responses appropriately reflect the intent of the question and the patient’s circumstances. Moreover, given that LLM-generated responses may inform real-world decision-making related to diagnosis, treatment, and medication prescribing, and even a single error may result in serious harm [104], the evaluation of clinical validity and logical rigor through expert judgment needs to be considered an important aspect. In contrast, in the public health domain, the understandability, practical applicability, and safe use of LLM-generated health information by laypeople were treated as core components of reliability. Laypeople need to understand, evaluate, and use health information when making health-related decisions [105], and prior literature has similarly emphasized that user-tailored communication and clear information delivery are important values of LLM applications in the public health domain [14,106]. Laypeople, particularly those with limited health literacy, may have difficulty evaluating the credibility of conflicting or potentially harmful health information, while inconsistent or confusing responses may lead to inappropriate decisions and actions [107,108]. Therefore, LLM reliability evaluation in the public health domain needs to consider user-centered aspects reflecting understandability, practical use, and safe use.

These findings highlight the limitations of applying a single uniform set of evaluation criteria to assess LLM reliability across different health care contexts. Reliability may not represent a universal fixed attribute and may instead be interpreted as a context-dependent concept, with evaluation criteria varying according to purpose, level of risk, and user characteristics. This interpretation is also consistent with previous research suggesting that the trustworthiness of medical AI should be understood in relation to its purpose of use and context of use [109,110]. Therefore, future evaluations of LLM reliability in health care may require greater consideration of domain-specific evaluation frameworks that reflect such contextual differences.

### Conceptual Inconsistency and the Need for Standardization

Beyond these domain-specific differences, conceptual inconsistency in evaluation indicators was identified as a common challenge across both domains. Even when the same indicator terminology was used, its meaning and scope of application varied considerably across studies. For example, indicators bearing the same label of “usefulness” were classified differently in this review as relevance, completeness, or clarity, depending on the definitions and evaluation criteria provided in each study [35,58,62,74,90]. In addition, some studies did not provide explicit operational definitions for the indicators used [96-100]. Such conceptual inconsistency in evaluation indicators may hinder direct comparisons across studies and limit the consistent accumulation of evidence on the reliability of health care LLMs [111,112]. Previous studies have also consistently noted the lack of consensus regarding evaluation dimensions and criteria in health care LLM evaluation and have emphasized that clearly defined evaluation dimensions and specific guidance for their application are important for improving the quality and interpretability of evaluations [15]. Therefore, future studies need to develop a standardized indicator framework that clearly specifies the conceptual definitions and scope of application of each evaluation indicator and apply it consistently across studies.

### Diversity of Evaluator Composition and Misalignment Between Evaluators and Evaluation Indicators

Across both domains, LLM reliability evaluation in health care has commonly relied on relatively small evaluator panels centered on clinicians. When evaluator panels are small, the influence of individual judgments on the overall findings is amplified, which may limit the stability and reproducibility of the results [113]. In the clinical domain, most evaluations were conducted by clinicians from a single specialty. Although this may ensure the expertise required for clinical judgment, it may also be associated with a risk of bias toward the perspective of a particular specialty, given that real-world clinical decision-making is inherently multidisciplinary and involves collaboration across professions [114]. In contrast, in the public health domain, mixed evaluator compositions involving nonclinicians, such as researchers, patients, and members of the general public, were more frequently observed. This may reflect efforts to incorporate the perspectives of diverse stakeholders.

However, regardless of domain, some studies showed suboptimal alignment between evaluator expertise and evaluation indicators [34,37,50,54]. For example, clinicians assessed user-centered indicators, such as comprehensibility, empathy, or patient appropriateness [50,54]. Such misalignment may increase interpretive discrepancies among evaluators and potentially undermine the consistency and validity of measurement [115]. Existing health care AI governance and evaluation frameworks have emphasized the importance of incorporating diverse stakeholder perspectives and multidisciplinary expertise into evaluation processes [101,116,117]. However, our findings suggest that evaluator diversity alone may be insufficient, and that appropriate alignment between evaluator expertise and evaluation indicators may represent an additional methodological consideration for ensuring high-quality reliability assessment.

### The Need for Clear Judgment Criteria and Evaluator Guidance

Likert scales and researcher-defined rubrics were the predominant evaluation approaches in both the clinical and public health domains. Likert scales facilitate comparisons across responses by quantifying subjective judgments, whereas researcher-defined rubrics allow detailed criteria to be tailored to the study purpose and the characteristics of the evaluation task [118,119]. However, some studies in this review identified differences in the interpretation of the same criteria, difficulty distinguishing between adjacent rating categories, and variation in assessments due to insufficient evaluator training as methodological limitations [36,52,53,71,85]. In particular, when the meaning and boundaries of rating categories are unclear, evaluators may assign scores based on criteria shaped by their own experience [120,121], which may hinder the accumulation of credible evidence in health care LLM evaluation [22]. In health information evaluation, standardized tools have been used to systematically assess information based on clearly defined evaluation domains and scoring criteria [122,123]. Future LLM reliability evaluations need to consider drawing on these evaluation approaches to break down broad evaluation concepts into specific judgment items and clearly define the meaning of each score level along with representative response examples. Evaluator training and practice evaluations need to be conducted before the formal evaluation so that evaluators can develop a shared understanding of the criteria, clearly distinguish between categories, and apply common judgment principles [124,125]. Clear judgment criteria and evaluator guidance may reduce unnecessary interpretive variation arising during the evaluation process and improve interrater agreement, thereby contributing to the methodological rigor and reproducibility of health care LLM reliability evaluations [126,127].

### Methodological Limitations of Human Evaluation and Future Considerations for LLM Reliability in Health Care

The methodological limitations of human evaluation identified in this review included evaluator subjectivity, the absence of standardized evaluation indicators, and limited evaluation scope and settings. In particular, human evaluation results may be influenced by subjective factors such as evaluators’ knowledge and experience and their interpretation of evaluation criteria [128]. In addition, differences in evaluation design, including the range of questions and cases, interaction formats, evaluation scales, and criteria, may also affect evaluation results [129]. These factors make it difficult to compare findings across studies and to accumulate consistent evidence on reliability [130]. Nevertheless, human evaluation serves as an important complementary approach to benchmark-based evaluation because it can assess the context and qualitative characteristics of responses that are difficult to capture using quantitative evaluation metrics alone [126,131,132]. Therefore, as emphasized in recent research on human evaluation of medical AI and LLMs, future human evaluations need to establish standardized frameworks that systematize evaluation indicators, criteria, and procedures while also considering evaluation designs that reflect the diverse situations and interactions of real-world health care settings [22,101]. Furthermore, as health care AI evolves toward agentic systems that integrate external tools, multistep reasoning, and autonomous decision-making capabilities, the scope of reliability evaluation may need to expand beyond the assessment of final responses alone [133-135]. Indeed, one previous study suggested that evaluating agentic AI may require additional evaluation dimensions, including planning, action execution, and error recovery, beyond those traditionally used for standalone LLMs [136]. The common evaluation dimensions and methodological considerations identified in this review provide a useful foundation for distinguishing reliability aspects that can be assessed using existing LLM-centered criteria from those requiring evaluation dimensions specific to agentic AI systems, and may contribute to the development of reliability indicators and evaluation frameworks for agentic AI in health care.

### Limitations

The findings of this review should be interpreted in light of several limitations. First, although preprints were included to capture recent developments in a rapidly evolving field, some findings may be less stable because these studies had not undergone formal peer review. Second, this review was conducted without formal protocol registration, which may limit the transparency and reproducibility of the review process. Third, some included studies did not provide sufficiently clear definitions of evaluation indicators or approaches, requiring researcher judgment during data charting, categorization, and interpretation. Although efforts were made to apply classification criteria consistently, some degree of subjectivity may have been involved in distinguishing between the clinical and public health domains and categorizing evaluation indicators, which may have influenced cross-study comparisons and interpretation of findings. Finally, studies included in this review were relatively concentrated in the public health domain, which may limit the extent to which characteristics of the clinical domain were represented. Accordingly, findings related to evaluation characteristics in the clinical domain should be interpreted with some caution. Nevertheless, to our knowledge, this is the first review to systematically examine the methodological structure of human evaluation–based LLM reliability assessment in health care, including evaluation indicators, evaluator composition, and evaluation approaches across clinical and public health domains.

### Conclusions

LLM reliability in health care is a context-dependent concept that cannot be adequately assessed using a single universal standard. Specifically, expert-centered clinical validity and logical rigor are key aspects of reliability in the clinical domain, whereas user-centered understandability, practical use, and safe use are key aspects in the public health domain. Nevertheless, common methodological challenges related to the design and conduct of human evaluations were identified across both domains. These issues may impede cross-study comparability and the systematic accumulation of reliability evidence. Therefore, it is important to establish a standardized reliability evaluation framework that reflects the characteristics and contexts of health care. Furthermore, as LLMs evolve into agentic AI systems, the scope of reliability evaluation needs to extend beyond final outputs to encompass reasoning processes and action selection. The evaluation dimensions and methodological considerations identified in this review are expected to provide a foundation for developing future reliability evaluation frameworks for LLMs and agentic AI in health care.

## Appendix

Multimedia Appendix 1 PRISMA-ScR (Preferred Reporting Items for Systematic Reviews and Meta-Analyses Extension for Scoping Reviews) checklist.

Multimedia Appendix 2 Search strategies for electronic databases.

Multimedia Appendix 3 Data chart of the included studies.

Multimedia Appendix 4 Studies contributing to each reported limitation category.

## Abbreviations

BLEU: bilingual evaluation understudy
GQS: Global Quality Scale
LLM: large language model
PEMAT: Patient Education Materials Assessment Tool
PRISMA-ScR: Preferred Reporting Items for Systematic Reviews and Meta-Analyses Extension for Scoping Reviews
PRISMA-S: Preferred Reporting Items for Systematic Reviews and Meta-Analyses Extension for Searching

## Data Availability

The datasets used and/or analyzed during this study are available from the corresponding author upon reasonable request.
