# Supplementary material for: The Reliability of Human Evaluation of Large Language Models in Health Care Settings: Scoping Review
Source: J Med Internet Res. 2026 Aug 19;28:e98184. doi: 10.2196/98184 (PMC13539180; doi:10.2196/98184)
Supplement: Supplementary file 2 [file jmir_v28i1e98184_app4.docx]

**Multimedia Appendix 4**

**Studies contributing to each reported limitation category.**

Only studies that explicitly reported limitations were included in this categorization; studies that did not specify any limitations were excluded [1-11].

**1. Evaluation Indicators & Approaches**

| **Limitation** | **Domain** | **Studies** |
| --- | --- | --- |
| **Subjectivity in Human Evaluation**  ● Subjective Judgment and Evaluator Bias  - Reliance on subjective judgment - Potential for evaluator bias  ● Variability and Limited Reliability of Evaluator Judgments  - Disagreement or inconsistency among evaluators  - Differences in the interpretation of evaluation criteria  - Lack of intra-rater or inter-rater reliability assessment | Clinical | [12-27] |
|  | Public Health | [28-52] |
| **Lack of Standardized Evaluation Metrics**  ● Lack of Standardized or Validated Evaluation Tools and Reference Standards  - Absence of standardized evaluation instruments or frameworks  - Use of nonvalidated or insufficiently validated assessment tools  - Absence or insufficient validation of a reference standard  ● Unclear or Insufficient Evaluation Criteria  - Unclear or insufficiently defined evaluation criteria  - Limited comprehensiveness of evaluation criteria  - Misalignment between evaluation criteria and evaluation tasks | Clinical | [12, 15, 16, 21, 27, 53-55] |
|  | Public Health | [30, 34, 35, 37, 39, 41, 43, 47, 51, 52, 56-61] |
| **Limitations of Rating Scale–Based Evaluation**  ● Ambiguity and Insufficient Granularity of Rating Scales  - Ambiguity in rating categories  - Difficulty distinguishing between adjacent scores  - Insufficient granularity of rating scales  ● Limitations in Reliability Assessment  - Restricted applicability of conventional inter-rater reliability statistics | Clinical | [19, 24, 26] |
|  | Public Health | [38, 51] |

**2. Evaluator Characteristics**

| **Limitation** | **Domain** | **Studies** |
| --- | --- | --- |
| **Insufficient Evaluator Sample Size**  ● Small or Insufficient Evaluator Samples  - Single evaluators or small evaluator panels  - Limited evaluator participation in specific assessments  ● Lack of Justification for Evaluator Sample Size  - Absence of sample size calculations or power | Clinical | [12, 14, 17-20, 22, 24, 27, 62] |
|  | Public Health | [30, 34, 37, 39, 41, 44, 49, 51, 57, 58, 61, 63-65] |
| **Restricted Evaluator Composition**  ● Limited Evaluator Diversity and Representativeness  - Limited professional or specialty diversity  - Limited institutional or geographic diversity  - Evaluators not representative of the intended user population  ● Exclusion of Relevant Stakeholders or End Users  - Exclusion of patients, caregivers, members of the general public, or health care team members  ● Limited Evaluator Independence or Expertise  - Reliance on internal research team members as evaluators  - Limited or variable evaluator expertis | Clinical | [14, 15, 17, 19, 20, 23, 27, 62] |
|  | Public Health | [28, 32, 35, 36, 39, 42, 44, 45, 48-50, 52, 57, 58, 63-65] |

**3. Evaluation Design**

| **Limitation** | **Domain** | **Studies** |
| --- | --- | --- |
| **Limited Evaluation Scope and Representativeness**  ● Narrow or Nonrepresentative Selection of Evaluation Cases  - Small or narrowly selected sets of questions, cases, scenarios, or tasks  - Lack of representative sampling of evaluation questions or cases  ● Limited Coverage of Domain Scope and Complexity  - Limited coverage of the target topic or clinical domain  - Limited ability to reflect the diversity and complexity of real-world cases | Clinical | [13, 53, 66, 67] |
|  | Public Health | [28, 29, 32, 34, 35, 59, 68, 69] |
| **Constrained Evaluation Settings**  ● Reliance on Static or Simulated Evaluation Settings  - Use of static, text-only, predefined, or structured scenarios  - Use of simulated rather than real-world cases  ● Limited Reflection of Real-World Interactions and Clinical Context  - Lack of dynamic or multi-turn conversational interaction  - Limited reflection of real-world clinical or patient settings  - Exclusion of clinical information such as imaging, physical examination findings, or detailed patient histories | Clinical | [13, 53, 66, 70] |
|  | Public Health | [28, 29, 35, 71] |
| **Sensitivity to Input Formulation**  ● Prompt-Related Variability and Limited Reproducibility  - Effects of prompt wording or phrasing  - Variability arising from dynamically formulated follow-up prompts  - Limited reproducibility due to variations in input formulation | Clinical | - |
|  | Public Health | [29] |

**Reference**

1. Chatzopoulos GS, Koidou VP, Tsalikis L, Kaklamanos EG. Evaluation of Large Language Model Performance in Answering Clinical Questions on Periodontal Furcation Defect Management. Dent J (Basel). 2025 Jun 18;13(6). PMID: 40559174. doi: 10.3390/dj13060271.

2. Saad M, Moqeet MA, Mansoor H, Khan S, Sharif R, Khan FU, et al. Evaluating the Efficacy of Artificial Intelligence-Driven Chatbots in Addressing Queries on Vernal Conjunctivitis. Cureus. 2025 Feb;17(2):e79688. PMID: 40161163. doi: 10.7759/cureus.79688.

3. Sivaramakrishnan G, Almuqahwi M, Ansari S, Lubbad M, Alagamawy E, Sridharan K. Assessing the power of AI: a comparative evaluation of large language models in generating patient education materials in dentistry. BDJ open. 2025;11(1):59. doi: 10.1038/s41405-025-00349-1.

4. Hassona Y, Alqaisi D, Al-Haddad A, Georgakopoulou EA, Malamos D, Alrashdan MS, et al. How good is ChatGPT at answering patients' questions related to early detection of oral (mouth) cancer? Oral Surg Oral Med Oral Pathol Oral Radiol. 2024 Aug;138(2):269-78. PMID: 38714483. doi: 10.1016/j.oooo.2024.04.010.

5. Temizsoy Korkmaz F, Ok F, Karip B, Keleş P. A structured evaluation of LLM-generated step-by-step instructions in cadaveric brachial plexus dissection. BMC Med Educ. 2025 Jul 1;25(1):903. PMID: 40598351. doi: 10.1186/s12909-025-07493-0.

6. Uz C, Umay E. "Dr ChatGPT": Is it a reliable and useful source for common rheumatic diseases? Int J Rheum Dis. 2023 Jul;26(7):1343-9. PMID: 37218530. doi: 10.1111/1756-185x.14749.

7. Vassis S, Powell H, Petersen E, Barkmann A, Noeldeke B, Kristensen KD, et al. Large-Language Models in Orthodontics: Assessing Reliability and Validity of ChatGPT in Pretreatment Patient Education. Cureus. 2024 Aug;16(8):e68085. PMID: 39347180. doi: 10.7759/cureus.68085.

8. Patel A, Ajumobi A. Evaluating the Reliability of OpenAI's ChatGPT-4 in Providing Pre-colonoscopy Patient Guidance. Cureus. 2025 Jun;17(6):e86512. PMID: 40698206. doi: 10.7759/cureus.86512.

9. Zhang S, Liau ZQG, Tan KLM, Chua WL. Evaluating the accuracy and relevance of ChatGPT responses to frequently asked questions regarding total knee replacement. Knee Surg Relat Res. 2024 Apr 2;36(1):15. PMID: 38566254. doi: 10.1186/s43019-024-00218-5.

10. Valentini M, Szkandera J, Smolle MA, Scheipl S, Leithner A, Andreou D. Artificial intelligence large language model ChatGPT: is it a trustworthy and reliable source of information for sarcoma patients? Frontiers in Public Health. 2024;12:1303319. doi: 10.3389/fpubh.2024.1303319.

11. Makrygiannakis MA, Giannakopoulos K, Kaklamanos EG. Evidence-based potential of generative artificial intelligence large language models in orthodontics: a comparative study of ChatGPT, Google Bard, and Microsoft Bing. Eur J Orthod. 2024 Apr 13. PMID: 38613510. doi: 10.1093/ejo/cjae017.

12. Magruder ML, Rodriguez AN, Wong JCJ, Erez O, Piuzzi NS, Scuderi GR, et al. Assessing Ability for ChatGPT to Answer Total Knee Arthroplasty-Related Questions. J Arthroplasty. 2024 Aug;39(8):2022-7. PMID: 38364879. doi: 10.1016/j.arth.2024.02.023.

13. Kotzur T, Singh A, Parker J, Peterson B, Sager B, Rose R, et al. Evaluation of a Large Language Model's Ability to Assist in an Orthopedic Hand Clinic. Hand (N Y). 2025 Sep;20(6):900-9. PMID: 38907651. doi: 10.1177/15589447241257643.

14. Ying L, Li S, Chen C, Yang F, Li X, Chen Y, et al. Screening/diagnosis of pediatric endocrine disorders through the artificial intelligence model in different language settings. Eur J Pediatr. 2024 Jun;183(6):2655-61. PMID: 38502320. doi: 10.1007/s00431-024-05527-1.

15. Liang Z, Wang M, Abdelatif NMN, Arunakul M, Borbon CAV, Chong KW, et al. Are Large Language Model-Based Chatbots Effective in Providing Reliable Medical Advice for Achilles Tendinopathy? An International Multispecialist Evaluation. Orthop J Sports Med. 2025 Apr;13(4):23259671251332596. PMID: 40322749. doi: 10.1177/23259671251332596.

16. Mykhalko Y, Dyditska S, Balatska L, Filak F, Rubtsova Y. AI-driven rehabilitation: evaluation of ChatGPT-4o for generating personalized physical rehabilitation plans in comorbid patients. Wiad Lek. 2025;78(4):753-9. PMID: 40367458. doi: 10.36740/WLek/203850.

17. Leypold T, Bahm J, Beier JP, Guillaume VG, Ammo T, Lauer H, et al. Evaluating ChatGPT o1's Capabilities in Peripheral Nerve Surgery: Advancing Artificial Intelligence in Clinical Practice. World Neurosurg. 2025 Apr;196:123753. PMID: 39924104. doi: 10.1016/j.wneu.2025.123753.

18. Cankurtaran RE, Polat YH, Aydemir NG, Umay E, Yurekli OT. Reliability and Usefulness of ChatGPT for Inflammatory Bowel Diseases: An Analysis for Patients and Healthcare Professionals. Cureus. 2023 Oct;15(10):e46736. PMID: 38022227. doi: 10.7759/cureus.46736.

19. Goodman RS, Patrinely JR, Stone CA, Jr., Zimmerman E, Donald RR, Chang SS, et al. Accuracy and Reliability of Chatbot Responses to Physician Questions. JAMA Netw Open. 2023 Oct 2;6(10):e2336483. PMID: 37782499. doi: 10.1001/jamanetworkopen.2023.36483.

20. Draschl A, Hauer G, Fischerauer SF, Kogler A, Leitner L, Andreou D, et al. Are ChatGPT's Free-Text Responses on Periprosthetic Joint Infections of the Hip and Knee Reliable and Useful? J Clin Med. 2023 Oct 20;12(20). PMID: 37892793. doi: 10.3390/jcm12206655.

21. Vaira LA, Lechien JR, Abbate V, Allevi F, Audino G, Beltramini GA, et al. Accuracy of ChatGPT-Generated Information on Head and Neck and Oromaxillofacial Surgery: A Multicenter Collaborative Analysis. Otolaryngol Head Neck Surg. 2024 Jun;170(6):1492-503. PMID: 37595113. doi: 10.1002/ohn.489.

22. Hoang T, Liou L, Rosenberg AM, Zaidat B, Duey AH, Shrestha N, et al. An analysis of ChatGPT recommendations for the diagnosis and treatment of cervical radiculopathy. J Neurosurg Spine. 2024 Sep 1;41(3):385-95. PMID: 38941643. doi: 10.3171/2024.4.Spine231148.

23. Fraile Navarro D, Coiera E, Hambly TW, Triplett Z, Asif N, Susanto A, et al. Expert evaluation of large language models for clinical dialogue summarization. Sci Rep. 2025 Jan 7;15(1):1195. PMID: 39774141. doi: 10.1038/s41598-024-84850-x.

24. Jin Z, Abola R, Bargnes V, 3rd, Tsivitis A, Rahman S, Schwartz J, et al. The utility of generative artificial intelligence Chatbot (ChatGPT) in generating teaching and learning material for anesthesiology residents. Front Artif Intell. 2025;8:1582096. PMID: 40469072. doi: 10.3389/frai.2025.1582096.

25. Rubinstein S, Mohsin A, Banerjee R, Ma W, Mishra S, Kwok M, et al. Summarizing clinical evidence utilizing large language models for cancer treatments: a blinded comparative analysis. Front Digit Health. 2025;7:1569554. PMID: 40364850. doi: 10.3389/fdgth.2025.1569554.

26. Balas M, Mandelcorn ED, Yan P, Ing EB, Crawford SA, Arjmand P. ChatGPT and retinal disease: a cross-sectional study on AI comprehension of clinical guidelines. Can J Ophthalmol. 2025 Feb;60(1):e117-e23. PMID: 39097289. doi: 10.1016/j.jcjo.2024.06.001.

27. Wilhelm TI, Roos J, Kaczmarczyk R. Large Language Models for Therapy Recommendations Across 3 Clinical Specialties: Comparative Study. J Med Internet Res. 2023 Oct 30;25:e49324. PMID: 37902826. doi: 10.2196/49324.

28. Lima HA, Trocoli-Couto P, Moazzam Z, Rocha LCD, Pagano A, Martins FF, et al. Quality assessment of large language models' output in maternal health. Sci Rep. 2025 Jul 2;15(1):22474. PMID: 40593918. doi: 10.1038/s41598-025-03501-x.

29. Birsel SE, Oto O, Görgün B, İnan İ H, Şeker A, İnan M. Is it a pediatric orthopaedic urgency or not? Can ChatGPT answer this question? J Orthop Surg Res. 2025 Jun 4;20(1):567. PMID: 40462187. doi: 10.1186/s13018-025-05981-z.

30. Lang S, Vitale J, Fekete TF, Haschtmann D, Reitmeir R, Ropelato M, et al. Are large language models valid tools for patient information on lumbar disc herniation? The spine surgeons' perspective. Brain and Spine. 2024;4:102804. doi: 10.1016/j.bas.2024.102804.

31. Wang Y, Liang L, Li R, Wang Y, Hao C. Comparison of the Performance of ChatGPT, Claude and Bard in Support of Myopia Prevention and Control. J Multidiscip Healthc. 2024;17:3917-29. PMID: 39155977. doi: 10.2147/jmdh.S473680.

32. Yan C, Li Z, Liang Y, Shao S, Ma F, Zhang N, et al. Assessing large language models as assistive tools in medical consultations for Kawasaki disease. Frontiers in Artificial Intelligence. 2025;8:1571503. doi: 10.3389/frai.2025.1571503.

33. Demir S. Evaluation of the reliability and readability of answers given by chatbots to frequently asked questions about endophthalmitis: A cross-sectional study on chatbots. Health Informatics J. 2024 Oct-Dec;30(4):14604582241304679. PMID: 39612357. doi: 10.1177/14604582241304679.

34. Yau JY, Saadat S, Hsu E, Murphy LS, Roh JS, Suchard J, et al. Accuracy of Prospective Assessments of 4 Large Language Model Chatbot Responses to Patient Questions About Emergency Care: Experimental Comparative Study. J Med Internet Res. 2024 Nov 4;26:e60291. PMID: 39496149. doi: 10.2196/60291.

35. Sezgin E, Jackson DI, Kocaballi AB, Bibart M, Zupanec S, Landier W, et al. Can Large Language Models Aid Caregivers of Pediatric Cancer Patients in Information Seeking? A Cross-Sectional Investigation. Cancer Med. 2025 Jan;14(1):e70554. PMID: 39776222. doi: 10.1002/cam4.70554.

36. Motegi M, Shino M, Kuwabara M, Takahashi H, Matsuyama T, Tada H, et al. Comparison of physician and large language model chatbot responses to online ear, nose, and throat inquiries. Sci Rep. 2025 Jul 1;15(1):21346. PMID: 40596359. doi: 10.1038/s41598-025-06769-1.

37. Yaş S, Yapar D, Yapar A, Özel T, Tokgöz MA, Baymurat AC, et al. Assessing the role of large language models in adolescent idiopathic scoliosis care: a comparison between ChatGPT and Google Gemini. Acta Orthop Traumatol Turc. 2025 Jul 18;59(4):222-9. PMID: 40728044. doi: 10.5152/j.aott.2025.25279.

38. Ah-Yan C, Boissonnault È, Boudier-Revéret M, Mares C. Impact of artificial intelligence in managing musculoskeletal pathologies in physiatry: a qualitative observational study evaluating the potential use of ChatGPT versus Copilot for patient information and clinical advice on low back pain. J Yeungnam Med Sci. 2025;42:11. PMID: 39610054. doi: 10.12701/jyms.2024.01151.

39. Lang S, Vitale J, Galbusera F, Fekete T, Boissiere L, Charles YP, et al. Is the information provided by large language models valid in educating patients about adolescent idiopathic scoliosis? An evaluation of content, clarity, and empathy : The perspective of the European Spine Study Group. Spine Deform. 2025 Mar;13(2):361-72. PMID: 39495402. doi: 10.1007/s43390-024-00955-3.

40. David D, Zloto O, Katz G, Huna-Baron R, Vishnevskia-Dai V, Armarnik S, et al. The use of artificial intelligence based chat bots in ophthalmology triage. Eye (Lond). 2025 Mar;39(4):785-9. PMID: 39592814. doi: 10.1038/s41433-024-03488-1.

41. Peled T, Sela HY, Weiss A, Grisaru-Granovsky S, Agrawal S, Rottenstreich M. Evaluating the validity of ChatGPT responses on common obstetric issues: Potential clinical applications and implications. Int J Gynaecol Obstet. 2024 Sep;166(3):1127-33. PMID: 38523565. doi: 10.1002/ijgo.15501.

42. Sahin Ozdemir M, Ozdemir YE. Comparison of the performances between ChatGPT and Gemini in answering questions on viral hepatitis. Sci Rep. 2025 Jan 11;15(1):1712. PMID: 39799203. doi: 10.1038/s41598-024-83575-1.

43. Kerkütlüoğlu M, Kaya E, Gökmen R. Trustworthiness, Value, Danger, and Readability of ChatGPT-Generated Responses to Health Questions Related to Pulmonary Arterial Hypertension. Cureus. 2024 Oct;16(10):e71472. PMID: 39544545. doi: 10.7759/cureus.71472.

44. Lang SP, Yoseph ET, Gonzalez-Suarez AD, Kim R, Fatemi P, Wagner K, et al. Analyzing Large Language Models' Responses to Common Lumbar Spine Fusion Surgery Questions: A Comparison Between ChatGPT and Bard. Neurospine. 2024 Jun;21(2):633-41. PMID: 38955533. doi: 10.14245/ns.2448098.049.

45. Piao Y, Chen H, Wu S, Li X, Li Z, Yang D. Assessing the performance of large language models (LLMs) in answering medical questions regarding breast cancer in the Chinese context. Digital Health. 2024;10:20552076241284771. doi: 10.1177/20552076241284771.

46. Mahedia M, Rohrich RN, Sadiq KO, Bailey L, Harrison LM, Hallac RR. Exploring the Utility of ChatGPT in Cleft Lip Repair Education. J Clin Med. 2025 Feb 4;14(3). PMID: 39941663. doi: 10.3390/jcm14030993.

47. Ye Y, Zheng ED, Lan QL, Wu LC, Sun HY, Xu BB, et al. Comparative evaluation of the accuracy and reliability of ChatGPT versions in providing information on Helicobacter pylori infection. Front Public Health. 2025;13:1566982. PMID: 40443929. doi: 10.3389/fpubh.2025.1566982.

48. Alabdulmohsen DM, Almahmudi MA, Alhashim JN, Almahdi MH, Alkishy EF, Almossabeh MJ, et al. Is ChatGPT a Reliable Source of Patient Information on Asthma? Cureus. 2024 Jul;16(7):e64114. PMID: 39119408. doi: 10.7759/cureus.64114.

49. King RC, Samaan JS, Yeo YH, Peng Y, Kunkel DC, Habib AA, et al. A Multidisciplinary Assessment of ChatGPT's Knowledge of Amyloidosis: Observational Study. JMIR Cardio. 2024 Apr 19;8:e53421. PMID: 38640472. doi: 10.2196/53421.

50. Wu Y, Zhang Z, Dong X, Hong S, Hu Y, Liang P, et al. Evaluating the performance of the language model ChatGPT in responding to common questions of people with epilepsy. Epilepsy Behav. 2024 Feb;151:109645. PMID: 38244419. doi: 10.1016/j.yebeh.2024.109645.

51. Ayık G, Ercan N, Demirtaş Y, Yıldırım T, Çakmak G. Evaluation of ChatGPT-4o's answers to questions about hip arthroscopy from the patient perspective. Jt Dis Relat Surg. 2025 Jan 2;36(1):193-9. PMID: 39719917. doi: 10.52312/jdrs.2025.1961.

52. Wright BM, Bodnar MS, Moore AD, Maseda MC, Kucharik MP, Diaz CC, et al. Is ChatGPT a trusted source of information for total hip and knee arthroplasty patients? Bone Jt Open. 2024 Feb 15;5(2):139-46. PMID: 38354748. doi: 10.1302/2633-1462.52.Bjo-2023-0113.R1.

53. Leypold T, Lingens LF, Beier JP, Boos AM. Integrating AI in Lipedema Management: Assessing the Efficacy of GPT-4 as a Consultation Assistant. Life (Basel). 2024 May 20;14(5). PMID: 38792666. doi: 10.3390/life14050646.

54. Naldi L, Bettoli V, Santoro E, Valetto MR, Bolzon A, Cassalia F, et al. Application of ChatGPT as a content generation tool in continuing medical education: acne as a test topic. Dermatol Reports. 2025 May 23;17(2). PMID: 39969058. doi: 10.4081/dr.2024.10138.

55. Liu X, Shi S, Zhang X, Gao Q, Wang W. The role of ChatGPT-4o in differential diagnosis and management of vertigo-related disorders. Sci Rep. 2025 May 28;15(1):18688. PMID: 40437044. doi: 10.1038/s41598-025-96309-8.

56. Büker M, Mercan G. Readability, accuracy and appropriateness and quality of AI chatbot responses as a patient information source on root canal retreatment: A comparative assessment. Int J Med Inform. 2025 Sep;201:105948. PMID: 40288015. doi: 10.1016/j.ijmedinf.2025.105948.

57. Roldan-Vasquez E, Mitri S, Bhasin S, Bharani T, Capasso K, Haslinger M, et al. Reliability of artificial intelligence chatbot responses to frequently asked questions in breast surgical oncology. J Surg Oncol. 2024 Aug;130(2):188-203. PMID: 38837375. doi: 10.1002/jso.27715.

58. Salmi L, Lewis DM, Clarke JL, Dong Z, Fischmann R, McIntosh EI, et al. A proof-of-concept study for patient use of open notes with large language models. JAMIA open. 2025;8(2):ooaf021. doi: 10.1093/jamiaopen/ooaf021.

59. Kamal AH. AI Chatbots in Pediatric Orthopedics: How Accurate Are Their Answers to Parents' Questions on Bowlegs and Knock Knees? Healthcare (Basel). 2025 May 27;13(11). PMID: 40508883. doi: 10.3390/healthcare13111271.

60. Kuşcu O, Pamuk AE, Sütay Süslü N, Hosal S. Is ChatGPT accurate and reliable in answering questions regarding head and neck cancer? Front Oncol. 2023;13:1256459. PMID: 38107064. doi: 10.3389/fonc.2023.1256459.

61. Aras N, Çalışkan N. Assessing the reliability and usefulness of ChatGPT responses on intermittent catheterization queries: A critical analysis. International Journal of Urological Nursing. 2024;18(3):e12428. doi: 10.1111/ijun.12428.

62. Leypold T, Schäfer B, Boos AM, Beier JP. Artificial Intelligence-Powered Hand Surgery Consultation: GPT-4 as an Assistant in a Hand Surgery Outpatient Clinic. J Hand Surg Am. 2024 Nov;49(11):1078-88. PMID: 39066762. doi: 10.1016/j.jhsa.2024.06.002.

63. Zalzal HG, Abraham A, Cheng J, Shah RK. Can ChatGPT help patients answer their otolaryngology questions? Laryngoscope Investigative Otolaryngology. 2024;9(1):e1193. doi: 10.1002/lio2.1193.

64. Lo Bianco G, Cascella M, Li S, Day M, Kapural L, Robinson CL, et al. Reliability, Accuracy, and Comprehensibility of AI-Based Responses to Common Patient Questions Regarding Spinal Cord Stimulation. J Clin Med. 2025 Feb 21;14(5). PMID: 40094896. doi: 10.3390/jcm14051453.

65. Calabrese G, Maselli R, Maida M, Barbaro F, Morais R, Nardone OM, et al. Unveiling the effectiveness of Chat-GPT 4.0, an artificial intelligence conversational tool, for addressing common patient queries in gastrointestinal endoscopy. Igie. 2025 Mar;4(1):21-5. PMID: 41648814. doi: 10.1016/j.igie.2025.01.012.

66. Gomez-Cabello CA, Borna S, Pressman SM, Haider SA, Forte AJ. Large Language Models for Intraoperative Decision Support in Plastic Surgery: A Comparison between ChatGPT-4 and Gemini. Medicina (Kaunas). 2024 Jun 8;60(6). PMID: 38929573. doi: 10.3390/medicina60060957.

67. Gumilar KE, Indraprasta BR, Faridzi AS, Wibowo BM, Herlambang A, Rahestyningtyas E, et al. Assessment of Large Language Models (LLMs) in decision-making support for gynecologic oncology. Comput Struct Biotechnol J. 2024 Dec;23:4019-26. PMID: 39610903. doi: 10.1016/j.csbj.2024.10.050.

68. Alqudah AA, Aleshawi AJ, Baker M, Alnajjar Z, Ayasrah I, Ta'ani Y, et al. Evaluating accuracy and reproducibility of ChatGPT responses to patient-based questions in Ophthalmology: An observational study. Medicine (Baltimore). 2024 Aug 9;103(32):e39120. PMID: 39121263. doi: 10.1097/md.0000000000039120.

69. Chen Y, Zhang S, Tang N, George DM, Huang T, Tang J. Using Google web search to analyze and evaluate the application of ChatGPT in femoroacetabular impingement syndrome. Front Public Health. 2024;12:1412063. PMID: 38883198. doi: 10.3389/fpubh.2024.1412063.

70. Kuerbanjiang W, Peng S, Jiamaliding Y, Yi Y. Performance Evaluation of Large Language Models in Cervical Cancer Management Based on a Standardized Questionnaire: Comparative Study. J Med Internet Res. 2025 Feb 5;27:e63626. PMID: 39908540. doi: 10.2196/63626.

71. Hack S, Alsleibi S, Saleh N, Alon EE, Rabinovics N, Remer E. Are chatbots a reliable source for patient frequently asked questions on neck masses? Eur Arch Otorhinolaryngol. 2025 Aug;282(8):4273-82. PMID: 40307608. doi: 10.1007/s00405-025-09433-6.
