# Supplementary material for: The Reliability of Human Evaluation of Large Language Models in Health Care Settings: Scoping Review
Source: J Med Internet Res. 2026 Aug 19;28:e98184. doi: 10.2196/98184 (PMC13539180; doi:10.2196/98184)
Supplement: Supplementary file 3 [file jmir_v28i1e98184_app2.doc]

### **Multimedia Appendix 2**

### **Search strategies for electronic databases.** Search run on 29 June 2026

| **Database** | **Where** | **Search command** | **Papers** |
| --- | --- | --- | --- |
| PubMed | MeSH, Title/Abstract | ("Large Language Models"[Mesh] OR "large language model"[Title/Abstract] OR "large language models"[Title/Abstract] OR LLM[Title/Abstract] OR LLMs[Title/Abstract] OR "generative language model"[Title/Abstract] OR "generative language models"[Title/Abstract] OR "neural language model"[Title/Abstract] OR "neural language models"[Title/Abstract] OR "generative AI"[Title/Abstract] OR "AI chatbot"[Title/Abstract] OR "AI chatbots"[Title/Abstract] OR chatbot[Title/Abstract] OR chatbots[Title/Abstract] OR "conversational AI"[Title/Abstract] OR "conversational agent"[Title/Abstract] OR "conversational agents"[Title/Abstract] OR ChatGPT[Title/Abstract] OR Claude[Title/Abstract] OR Gemini[Title/Abstract]) AND ("Medicine"[Mesh] OR "Clinical Medicine"[Mesh] OR "Public Health"[Mesh] OR "Patients"[Mesh] OR healthcare[Title/Abstract] OR "health care"[Title/Abstract] OR medical[Title/Abstract] OR medicine[Title/Abstract] OR clinical[Title/Abstract] OR patient[Title/Abstract] OR patients[Title/Abstract] OR "public health"[Title/Abstract]) AND (evaluation[Title/Abstract] OR evaluate[Title/Abstract] OR evaluated[Title/Abstract] OR evaluating[Title/Abstract] OR assessment[Title/Abstract] OR assess[Title/Abstract] OR assessed[Title/Abstract] OR assessing[Title/Abstract] OR "human evaluation"[Title/Abstract] OR "qualitative evaluation"[Title/Abstract] OR "expert evaluation"[Title/Abstract] OR "human assessment"[Title/Abstract] OR "expert assessment"[Title/Abstract]) AND (reliability[Title/Abstract] OR reliable[Title/Abstract] OR trustworthiness[Title/Abstract] OR trustworthy[Title/Abstract] OR trust[Title/Abstract] OR trusted[Title/Abstract] OR credibility[Title/Abstract]) | 1,597 |
| Web of Science | TS (topic) | TS=("large language model" OR "large language models" OR LLM OR LLMs OR "generative language model" OR "generative language models" OR "neural language model" OR "neural language models" OR "generative AI" OR "AI chatbot" OR "AI chatbots" OR chatbot OR chatbots OR "conversational AI" OR "conversational agent" OR "conversational agents" OR ChatGPT OR Claude OR Gemini) AND TS=(medicine OR healthcare OR "health care" OR medical OR clinical OR patient OR patients OR "public health") AND TS=(evaluation OR evaluate OR evaluated OR evaluating OR assessment OR assess OR assessed OR assessing OR "human evaluation" OR "qualitative evaluation" OR "expert evaluation" OR "human assessment" OR "expert assessment") AND TS=(reliability OR reliable OR trustworthiness OR trustworthy OR trust OR trusted OR credibility) | 1,849 |
| Cochrane Library | Title, Abstract, Keyword | ("large language model" OR "large language models" OR LLM OR LLMs OR "generative language model" OR "generative language models" OR "neural language model" OR "neural language models" OR "generative AI" OR "AI chatbot" OR "AI chatbots" OR chatbot OR chatbots OR "conversational AI" OR "conversational agent" OR "conversational agents" OR ChatGPT OR Claude OR Gemini) AND (medicine OR healthcare OR "health care" OR medical OR clinical OR patient OR patients OR "public health") AND (evaluation OR evaluate OR evaluated OR evaluating OR assessment OR assess OR assessed OR assessing OR "human evaluation" OR "qualitative evaluation" OR "expert evaluation" OR "human assessment" OR "expert assessment") AND (reliability OR reliable OR trustworthiness OR trustworthy OR trust OR trusted OR credibility) | 53 |
| CINAHL | Headings, Title/Abstract | (MH "Artificial Intelligence" OR MH "Natural Language Processing" OR TI "large language model" OR AB "large language model" OR TI "large language models" OR AB "large language models" OR TI LLM OR AB LLM OR TI LLMs OR AB LLMs OR TI "generative language model" OR AB "generative language model" OR TI "generative language models" OR AB "generative language models" OR TI "neural language model" OR AB "neural language model" OR TI "neural language models" OR AB "neural language models" OR TI "generative AI" OR AB "generative AI" OR TI "AI chatbot" OR AB "AI chatbot" OR TI "AI chatbots" OR AB "AI chatbots" OR TI chatbot OR AB chatbot OR TI chatbots OR AB chatbots OR TI "conversational AI" OR AB "conversational AI" OR TI "conversational agent" OR AB "conversational agent" OR TI "conversational agents" OR AB "conversational agents" OR TI ChatGPT OR AB ChatGPT OR TI Claude OR AB Claude OR TI Gemini OR AB Gemini) AND (MH "Health Care" OR MH "Medicine" OR MH "Public Health" OR MH "Patients" OR TI healthcare OR AB healthcare OR TI "health care" OR AB "health care" OR TI medical OR AB medical OR TI medicine OR AB medicine OR TI clinical OR AB clinical OR TI patient OR AB patient OR TI patients OR AB patients OR TI "public health" OR AB "public health") AND (MH "Evaluation" OR TI evaluation OR AB evaluation OR TI evaluate OR AB evaluate OR TI evaluated OR AB evaluated OR TI evaluating OR AB evaluating OR TI assessment OR AB assessment OR TI assess OR AB assess OR TI assessed OR AB assessed OR TI assessing OR AB assessing OR TI "human evaluation" OR AB "human evaluation" OR TI "qualitative evaluation" OR AB "qualitative evaluation" OR TI "expert evaluation" OR AB "expert evaluation" OR TI "human assessment" OR AB "human assessment" OR TI "expert assessment" OR AB "expert assessment") AND (TI reliability OR AB reliability OR TI reliable OR AB reliable OR TI trustworthiness OR AB trustworthiness OR TI trustworthy OR AB trustworthy OR TI trust OR AB trust OR TI trusted OR AB trusted OR TI credibility OR AB credibility) | 548 |
| Google Scholar | Not applicable | site:arxiv.org ("large language model" OR "large language models" OR LLM OR LLMs OR "generative language model" OR "generative language models" OR "neural language model" OR "neural language models" OR "generative AI" OR "AI chatbot" OR "AI chatbots" OR chatbot OR chatbots OR "conversational AI" OR "conversational agent" OR "conversational agents" OR ChatGPT OR Claude OR Gemini) (medicine OR healthcare OR "health care" OR medical OR clinical OR patient OR patients OR "public health") (evaluation OR evaluate OR assessed OR assessment OR "human evaluation" OR "qualitative evaluation" OR "expert evaluation" OR "human assessment" OR "expert assessment") (reliability OR reliable OR trustworthiness OR trustworthy OR trust OR trusted OR credibility) | 300 |
|  |  | **TOTAL** | 4,347 |

ss
